# Diagnostic Value of miR-103 in Patients with Sepsis and Noninfectious SIRS and Its Regulatory Role in LPS-Induced Inflammatory Response by Targeting TLR4

**DOI:** 10.1155/2020/2198308

**Published:** 2020-05-13

**Authors:** Min Yang, Li Zhao, Mingyan Sun

**Affiliations:** ^1^Department of Infectious Diseases, Affiliated Hospital of Weifang Medical University, Weifang, Shandong 261031, China; ^2^Department of Infectious Diseases, Yidu Central Hospital of Weifang, Weifang, Shandong 262500, China; ^3^Clinical Laboratory, Affiliated Hospital of Weifang Medical University, Weifang, Shandong 261031, China

## Abstract

**Background:**

Sepsis is a life-threatening condition and a systemic inflammatory response syndrome (SIRS) driven by infection. This study aimed at investigating the expression of microRNA-103 (miR-103) in sepsis patients, evaluating its diagnostic value, and exploring the regulatory effect of miR-103 on LPS-induced inflammation in monocytes.

**Methods:**

Expression of miR-103 was measured using quantitative real-time PCR. A receiver operating characteristics curve was plotted to evaluate the diagnostic vale of miR-103. Serum and cell supernatant levels of proinflammatory cytokines were analyzed using ELISA. The interaction between miR-103 and Toll-like receptors 4 (TLR4) was analyzed using luciferase reporter assay. The effect of miR-103 on inflammation was examined in LPS-treated monocytes.

**Results:**

Serum expression of miR-103 was decreased in noninfectious SIRS and sepsis patients compared with healthy controls, and the lowest expression value was observed in sepsis patients (all *P* < 0.05). Serum levels of miR-103 have considerable diagnostic accuracy in distinguishing sepsis patients from SIRS patients and healthy controls. A negative correlation was found between miR-103 and inflammatory responses in sepsis patients. TLR4 was demonstrated to be a direct target of miR-103 and was negatively regulated by miR-103 in monocytes. The promoted inflammatory responses by LPS in monocytes were reversed by the overexpression of miR-103.

**Conclusion:**

All the data revealed that serum decreased miR-103 in sepsis patients serves as a promising noninvasive diagnostic biomarker and may be involved in the pathogenesis of sepsis by regulating inflammatory responses via targeting TLR4.

## 1. Introduction

Sepsis is a kind of systemic inflammatory response syndrome (SIRS) driven by infection [1]. It is considered a life-threatening condition caused by the abnormal host response to infection and a leading cause of deaths in intensive care units (ICUs) [2]. The statistics revealed that there are approximately 250,000 new deaths occurred in ICUs due to sepsis in the United States [3]. The incidence of sepsis is increasing in elderly people, and about 60% sepsis patients in ICUs are older than 65 years old [4]. Currently, the gold standard for sepsis diagnosis is blood microbiological culture analysis, but this method spends more time to get the examination results than other molecular biomarkers, such as procalcitonin (PCT) and C-reactive protein (CRP) [5]. However, these frequently used molecules lack specificity in distinguishing sepsis cases from noninfectious diseases [6]. Therefore, novel biomarkers with high sensitivity and specificity are necessary for the early diagnosis of sepsis.

MicroRNAs (miRNAs) are a group of small noncoding RNAs with critical regulatory roles in gene expression at posttranscriptional levels [7]. Numerous studies have provided evidence for miRNAs to be involved in the regulation of cellular processes and to serve as promising diagnostic and prognostic biomarkers in various diseases [8, 9]. A characteristic of miRNAs to be stably detected from serum or plasma samples contributes miRNAs to be a kind of a good diagnostic tool [10]. In patients with sepsis, some miRNAs have been identified to be candidate diagnostic biomarkers, such as miR-155-5p and miR-133a-3p [11]. Toll-like receptors (TLRs) have been reported to serve as important roles in sepsis [12]. TLR4 is a widely investigated TLRs in sepsis, which is a key molecule in the innate immune system and the development of sepsis [13]. Some potential therapeutic methods have been demonstrated in sepsis treatment by inhibiting the TLR4 signaling [14]. In this study, we found a complementary sequence of miR-103 at the 3′-untranslated region (3′-UTR) of TLR4. Previous studies have demonstrated the regulatory effects of miR-103 on inflammatory responses in some human diseases, such as Alzheimer's disease [15] and atherosclerosis [16]. Of note, a study by Huang et al. investigated the miRNA expression profiles in neonatal sepsis using neonatal monocytes, which are key leukocytes that link innate to adaptive immunity, and found that miR-103 was decreased in LPS-treated cells [17]. The results of the previous studies remind us that miR-103 might also be involved in the development of sepsis.

To improve the diagnosis and treatment of sepsis, this study aimed at investigating the expression of miR-103 in sepsis patients and evaluating the diagnostic potential of miR-103 in distinguishing sepsis patients from noninfectious SIRS patients and healthy individuals. In addition, this study used LPS-treated monocytes to explore the effect of miR-103 on inflammatory responses.

## 2. Materials and Methods

### 2.1. Patients and Sample Collection

A total of 108 sepsis patients and 89 age- and gender-matched noninfectious SIRS patients were recruited in this study from the ICU of Affiliated Hospital of Weifang Medical University between 2014 and 2017. The diagnosis of patients was performed on the basis of blood microbiological culture results according to the criteria of the American College of Chest Physicians/Society of Critical Care Medicine [18]. The disease severity of the sepsis patients was defined by the sepsis-related organ failure assessment (SOFA) score [19] and the acute physiology and chronic health evaluation (APACHE) II score [20] obtained on day 1 after ICU admission. In addition, 68 healthy volunteers were enrolled in this study as healthy controls, and no statistical significance was presented between the healthy individuals and the sepsis and SIRS patients. Blood samples were collected from the participants at the time of initial diagnosis, and serum was isolated from the blood by centrifugation and stored at -80°C. The experimental protocols of this study were approved by the Ethics Committee of Affiliated Hospital of Weifang Medical University, and a written informed consent was obtained from each participant.

### 2.2. Monocytes Collection and LPS Treatment

The blood samples collected from sepsis patients were settled by 4.5% dextran 500 (1 : 5; Amersham Biosciences, Piscataway, NJ, USA), and the leukocytes were separated from the red blood cells. Monocytes were isolated using density gradient centrifugation with Ficoll-Paque (Amersham Pharmacia, Biotech AB, Uppsala, Sweden) as previously described [21]. After the isolation, the monocytes were cultured in RPMI-1640 medium containing 10% FBS at 37°C in a humidified incubator with 5% CO_2_. To simulate the inflammatory response progression of sepsis, 100 ng/mL lipopolysaccharide (LPS; Sigma-Aldrich, Louis, MO, USA) was used to stimulate monocytes to induce excessive inflammatory responses.

### 2.3. Cell Transfection

To regulate the expression of miR-103 in monocytes, the cells were transfected with miR-103 mimic, miR-103 inhibitor, or the negative controls (mimic NC and inhibitor NC) (GenePharma, Shanghai, China) by Lipofectamine 2000 (Thermo Fisher Scientific, Waltham, MA, USA) following the manufacturers' protocol. This experiment was repeated three times.

### 2.4. RNA Extraction and Quantitative Real-Time PCR (qRT-PCR)

Total RNA was extracted from monocytes and serum samples collected from 108 sepsis patients, 89 noninfectious SIRS patients, and 68 healthy controls by TRIzol reagent (Invitrogen, Carlsbad, CA, USA), and a NanoDrop 2000 (Thermo Fisher Scientific, Waltham, MA, USA) was used to evaluate the concentration and purity of RNA. The obtained RNA was reversely transcribed into cDNA by a PrimeScript RT reagent kit (TaKaRa, Shiga, Japan) following the manufacturer's instruction. The relative expression of miR-103 and mRNA of TLR4 was examined using qRT-PCR, which was conducted by a SYBR green I Master Mix kit (Invitrogen, Carlsbad, CA, USA) and a 7300 Real-Time PCR System (Applied Biosystems, USA). Each sample was examined for at least three times. The final relative expression values were calculated using the 2^−*ΔΔ*Ct^ method and normalized to U6 or GAPDH.

### 2.5. Enzyme-Linked Immune Sorbent Assay (ELISA)

This study measured the serum or cell supernatant levels of proinflammatory cytokines to reflect the inflammatory response status. The levels of IL-1*β*, IL-6, and TNF-*α* were measured using an ELISA kit (Bioscience, San Diego, CA, USA) following the protocols of manufacturers, and the optical density (OD) at 450 nm was read by a microplate reader (Bio-Rad, Hercules, CA, USA). This experiment was performed in triplicate.

### 2.6. Luciferase Reporter Assay

This study used miRanda (http://www.microrna.org/microrna/home.do) to predict that TLR4 contains a complementary sequence of miR-103. A subsequent luciferase reporter assay was performed to verify the interaction between miR-103 and TLR4. The wild type (WT) and mutant type (MUT) 3′-UTRs of TLR4 were separately cloned in the pGL3 basic vector (Promega, Madison, WI), and the combined vectors were cotransfected into monocytes with miR-103 mimic, miR-103 inhibitor, or the NCs using Lipofectamine 2000 (Thermo Fisher Scientific, Waltham, MA, USA). The luciferase activity in each group was measured using a Dual-Luciferase Reporter Assay System (Promega), and this assay was repeated three times.

### 2.7. Statistical Analysis

The data obtained from this study was expressed as mean ± SD and analyzed using the SPSS 18.0 software (SPSS Inc., Chicago, IL) and GraphPad Prism 5.0 software (GraphPad Software, Inc., USA). Differences between groups were compared using Student's *t* test, one-way ANOVA, or Chi-squared test. Correlations between indicators are assessed using Pearson's correlation coefficient. A receiver operating characteristics (ROC) curve was performed to evaluate the diagnostic performance of miR-103. All the analyses were repeated at least three times. A difference with a *P* < 0.05 indicated statistically significant.

## 3. Results

### 3.1. Serum Expression of miR-103 in Sepsis Patients

**Figure 1:**
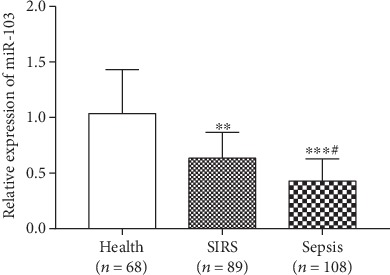
Expression of serum miR-103 in sepsis patients (*n* = 108), noninfectious SIRS patients (*n* = 89), and healthy controls (*n* = 68). ^∗∗^*P* < 0.01 and ^∗∗∗^*P* < 0.001 compared with the healthy group; ^#^*P* < 0.05 compared with the SIRS group.

According to qRT-PCR, the serum expression of miR-103 was examined. As shown in Figure 1, the expression of miR-103 was decreased in both the noninfectious SIRS patients (*n* = 89) and sepsis patients (*n* = 108) compared with the healthy controls (*n* = 68) (both *P* < 0.01). In addition, a lower expression of miR-103 was observed in sepsis patients when compared to the expression results in the noninfectious SIRS patients (*P* < 0.05).

### 3.2. Correlation between miR-103 and Clinicopathological Characteristics of Patients with Sepsis

This study summarized the clinicopathological features of the sepsis patients, including body mass index (BMI), white blood cell (WBC), CRP, PCT, APACHE II score, and SOFA score. To analyze the potential role of miR-103 in sepsis development, the correlation between miR-103 and the clinical data of sepsis patients was assessed. In Table 1, we found that the expression of miR-103 was negatively correlated with the WBC, CRP, PCT, APACHE II score, and SOFA score of sepsis patients (all *P* < 0.001), while there was no significant correlation between miR-103 and BMI (*P* > 0.05).

### 3.3. Diagnostic Performance of Serum Levels of miR-103

**Figure 2:**
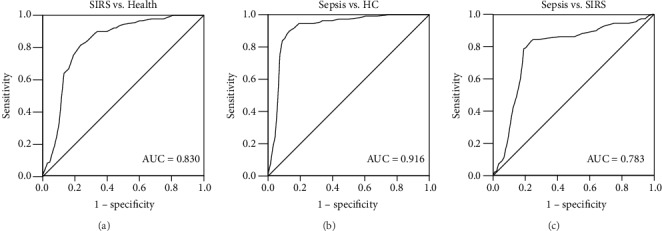
ROCs for SIRS and sepsis patients based on serum miR-103 expression levels. (a) A ROC for the differentiation between noninfectious SIRS patients (*n* = 89) from healthy individuals (*n* = 68). (b) A ROC in distinguishing sepsis patients (*n* = 108) from healthy individuals (*n* = 68). (c) A ROC for the differentiation between sepsis cases (*n* = 108) from noninfectious SIRS (*n* = 89). AUC: area under the curve.

Given the deregulated expression of miR-103 in SIRS patients and sepsis patients compared with healthy individuals, the diagnostic potential of miR-103 was further evaluated. As shown in Figure 2(a), the area under the curve (AUC) was 0.830 for miR-103 to distinguish SIRS patients from healthy controls. The results shown in Figure 2(b) revealed the high diagnostic accuracy of miR-103 in the distinguishing between sepsis patients from healthy individuals, as evidenced by the AUC of 0.916 and sensitivity and specificity of 89.8% and 88.2% at a cutoff value of 0.685. Moreover, the diagnostic performance of miR-103 in the differentiation of sepsis from SIRS was also examined. As shown in Figure 2(c), the AUC was 0.783 with a sensitivity and specificity of 83.3% and 76.4% at a cutoff value of 0.525, indicating the diagnostic value of miR-103 in the screening of sepsis patients from noninfectious SIRS patients.

### 3.4. Correlation of miR-103 with Inflammation of Sepsis Patients

This study analyzed the correlation of miR103 with inflammatory responses in sepsis patients. The results listed in Table 2 indicated that serum levels of miR-103 were negatively correlated with the levels of IL-1*β* (*r* = −0.691, *P* < 0.001), IL-6 (*r* = −0.725, *P* < 0.001), and TNF-*α* (*r* = −0.654, *P* < 0.001) in patients with sepsis.

### 3.5. Expression of miR-103 and TLR4 in LPS-Treated Monocytes

**Figure 3:**
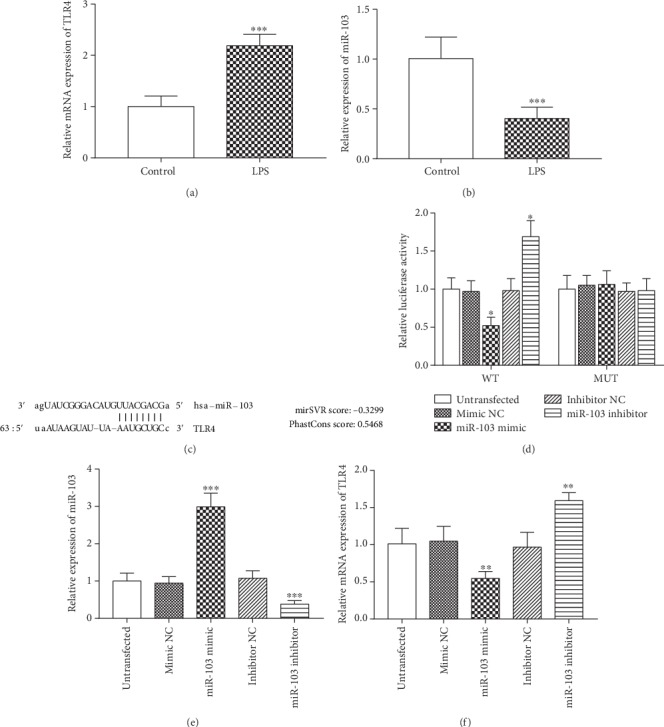
miR-103 directly inhibited the expression of TLR4 in monocytes under LPS treatment. (a, b) Relative expression of TLR3 mRNA (a) and miR-103 (b) in monocytes treated with LPS (^∗∗∗^*P* < 0.001). (c) The predictive binding site of miR-103 on the 3′-UTR of TLR4. (d) Luciferase activity results to confirm the interaction of miR-103 and TLR4 (^∗^*P* < 0.05 compared with the untransfected group). (e) Cell transfection efficiency examination results (^∗∗∗^*P* < 0.001 compared with the untransfected group). (f) Inhibiting effect of miR-103 on the mRNA expression of TLR4 in LPS-treated monocytes (^∗∗^*P* < 0.01 compared with the untransfected group). The data were collected from the experiments that were repeated at least three times.

The collected monocytes were treated with LPS to simulate the pathogenesis of sepsis. The expression of miR-103 and TLR4 was measured by qRT-PCR in monocytes stimulated by LPS. The results shown in Figures 3(a) and 3(b) indicated that the mRNA expression of TLR4 was as expected to be increased in the LPS-treated group, while the relative expression of miR-103 was decreased in monocytes treated with LPS (both *P* < 0.001). A complementary sequence of miR-103 was predicted at the 3′-UTR of TLR4 (Figure 3(c)), and the subsequent luciferase reporter assay was conducted to confirm their interaction. As shown in Figure 3(d), the relative luciferase activity was significantly inhibited by the overexpression of miR-103 but was promoted by the reduction of miR-103 in the WT group (both *P* < 0.05). No significant changes were found in the luciferase activity in MUT group (*P* > 0.05). Furthermore, the regulatory effect of miR-103 on the expression of TLR4 was checked by regulating the expression of miR-103 in monocytes. The cell transfection efficiency results showed that the expression of miR-103 was successfully upregulated by the miR-103 mimic and was downregulated by the miR-103 inhibitor (both *P* < 0.001, Figure 3(e)). The mRNA expression of TLR4 results shown in Figure 3(f) revealed that the upregulation of miR-103 could inhibit, while the downregulation of miR-103 could promote, TLR4 expression in the LPS-treated monocytes (all *P* < 0.01).

### 3.6. Effect of miR-103 on Inflammatory Response in LPS-Treated Monocytes

**Figure 4:**
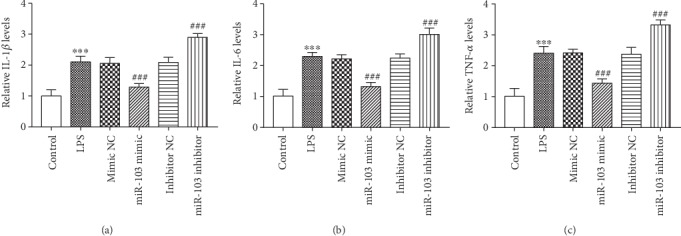
Effect of miR-103 on inflammation in LPS-treated monocytes. The levels of IL-1*β* (a), IL-6 (b), and TNF-*α* (c) in cell culture supernatants were inhibited by the overexpression but were enhanced by the knockdown of miR-103. The data were collected from the experiments that were repeated at least three times. ^∗∗∗^*P* < 0.001 compared with the control group; ^###^*P* < 0.001 compared with the LPS group.

After the in vitro manipulation of miR-103, the changes in inflammatory responses in monocytes treated by LPS were further investigated. As shown in Figure 4, we observed that the increased levels of IL-1*β*, IL-6, and TNF-*α* induced by LPS stimulation were all reversed by the overexpression of miR-103, while aggravated by the knockdown of miR-103 in monocytes (all *P* < 0.01).

## 4. Discussion

Dysregulation of miRNAs have attracting increasing attention for their critical roles in the pathogenesis of various human diseases, including inflammatory diseases such as SIRS [22]. Sepsis is considered a SIRS caused by infection, and the understanding about the immunopathology of SIRS and sepsis is important for the development of novel diagnostic and therapeutic approaches [23]. Currently, some aberrantly expressed miRNAs have been identified in sepsis. For example, the decreased miR-23b expression detected in the peripheral blood mononuclear cells of sepsis patients was negatively correlated with inflammatory response and could alleviate the LPS-stimulated inflammatory cytokines [24]. The decreased expression of miR-21 was reported in patients with sepsis and exhibited a good value to predict sepsis risk and thus was determined as a candidate biomarker for the development and progression of sepsis [25]. In patients collected from ICU, a low-level serum of miR-143 was indicated to be an indicator to predict the onset of sepsis and disease severity [26]. These previous results indicate the critical roles of deregulated miRNAs in the pathogenesis of sepsis.

A study by Huang et al. reported the dysregulation of miR-103 in neonatal sepsis, and that miR-103 expression was reduced in neonatal monocytes treated by LPS [17]. In this study, we first investigated the expression of miR-103 in adult sepsis and found that serum expression of miR-103 was decreased in noninfectious SIRS and sepsis patients compared with healthy individuals, and that a lower miR-103 level was demonstrated in sepsis than that in the noninfectious SIRS. In addition, the significant association between miR-103 and the clinical characteristics, including WBC, CRP, PCT, APACHE II score, and SOFA score, was found in sepsis patients, implying that miR-103 might be involved in the development and progression of sepsis.

miRNAs can be easily detected from serum and plasma samples, making them a group of good diagnostic tools in various diseases [27]. Guo et al. provided evidence for the decreased serum miR-495 as a diagnostic biomarker to screen sepsis patients from healthy population [28]. Lan et al. reported that serum miR-155-5p and miR-133a-3p levels were increased in sepsis and related with disease severity, which served as candidate biomarkers for the diagnosis of sepsis [11]. The downregulated miR-146a expression in sepsis patients was determined to be a diagnostic biomarker in distinguishing sepsis patients from non-sepsis-SIRS patients [6]. Considering the lowest miR-103 expression in sepsis patients compared with noninfectious SIRS patients and healthy volunteers, the diagnostic potential of miR-103 was further evaluated. The ROC analysis data indicated that the decreased expression of miR-103 had relatively high diagnostic accuracy in differentiation of sepsis and noninfectious SIRS from healthy individuals. It is worth noting that the low expression of miR-103 also had considerable diagnostic value for distinguishing sepsis patients from noninfectious SIRS patients. Since sepsis is a life-threatening condition, early diagnosis, especially the screening of sepsis from non-sepsis-SIRS, is of great importance for timely treatment. Our results provided evidence for miR-103 to serve as a candidate biomarker to screen sepsis cases from SIRS patients.

Previous studies have showed that miR-103 plays an important regulatory role in inflammatory responses, such as the inhibiting effect of miR-103 on inflammation in obesity and metabolic syndrome-related disorders [29], chronic obstructive pulmonary disease [30], and Alzheimer's disease [15]. In the present study, the serum miR-103 was found to be negatively correlated with inflammatory cytokine levels in sepsis patients. Moreover, the overexpression of miR-103 could inhibit the LPS-induced inflammatory response in monocytes. These results suggested that miR-103 might be a potential therapeutic target of sepsis by ameliorating the pathologic inflammation. TLR4 is one of the widely studied TLRs, which acts as a key molecule in the regulation of inflammatory response in sepsis [13]. The 3′-UTR of TLR4 contained a complementary sequence of miR-103, and we demonstrated that miR-103 could directly bind to the 3′-UTR of TLR4 and negatively regulated TLR4 mRNA expression in monocytes. The important role of TLR4 in sepsis combined with our study results led us to deduce that miR-103 might inhibit the inflammatory response of sepsis by targeting TLR4.

In conclusion, this study demonstrated that serum having decreased miR-103 serves as a candidate diagnostic biomarker to screen sepsis patients from noninfectious SIRS patients and healthy population. The overexpression of miR-103 may be a promising therapeutic target of sepsis by attenuating inflammatory response through the TLR4 signaling. Although this study provides a novel insight into the diagnosis and therapy of sepsis, the clinical significance of serum miR-103 and the functional role of miR-103 in sepsis are needed to be confirmed in further studies.

## Figures and Tables

**Table 1:** Correlation of miR-103 with clinical characteristics of 108 sepsis patients.

Indicators	miR-103	
	Correlation coefficient (*r*)	*P* value
BMI	0.181	0.061
WBC	-0.818	<0.001
CRP	-0.736	<0.001
PCT	-0.674	<0.001
APACHE II score	-0.767	<0.001
SOFA score	-0.680	<0.001

BMI: body mass index; WBC: white blood cell; CRP: C-reactive protein; PCT: procalcitonin; APACHE: acute physiology and chronic health evaluation; SOFA: sepsis-related organ failure assessment.

**Table 2:** Correlation of miR-103 with proinflammatory cytokines in 108 sepsis patients.

Indicators	miR-103	
	Correlation coefficient (*r*)	*P* value
IL-1*β*	-0.691	<0.001
IL-6	-0.725	<0.001
TNF-*α*	-0.654	<0.001

IL: interleukin; TNF: tumor necrosis factor.

## Data Availability

All data generated or analyzed during this study are included in this published article.
